# A Comparative Evaluation of Functional and Physiological Impression Techniques for Bilateral Distal Extension Removable Partial Dentures

**DOI:** 10.7759/cureus.105598

**Published:** 2026-03-21

**Authors:** Arvind K Singh, Shruti S Grover, Amrita Jayaswal, Shalini Pandey, Joy K Baishya

**Affiliations:** 1 Department of Prosthodontics, Chandra Dental College and Hospital, Barabanki, IND

**Keywords:** altered cast technique, functional reline method, hindels method, prosthodontics, removable partial denture, selective pressure method, tissue displacement

## Abstract

Aim and objectives

To compare the tissue displacement produced by the Hindels impression method, the selective pressure method, and the functional reline method in the anterior, middle, and posterior regions of bilateral distal extension ridges. Additionally, the study aims to identify the impression technique that provides optimal tissue recording for distal extension removable partial denture support.

Materials and methods

A patient with bilaterally missing mandibular posterior teeth was selected for the study. Five definitive casts were fabricated using each of the three impression techniques. Tissue displacement in the anterior, middle, and posterior regions was measured using a digital vernier caliper. The collected data were subjected to statistical analysis to evaluate intra-group and inter-group differences among the three techniques.

Results

Within each technique, tissue displacement values increased from the anterior to the posterior regions; however, these differences were not statistically significant (P > 0.05). Inter-group comparison demonstrated that both the Hindels and selective pressure methods produced significantly greater tissue displacement than the functional reline method across all regions (P < 0.001). No statistically significant difference was observed between the Hindels and selective pressure methods (P > 0.05). The overall mean tissue displacement was highest with the selective pressure method, followed closely by the Hindels method, and lowest with the functional reline method, indicating better functional tissue recording for distal extension removable partial denture support.

Conclusion

The Hindels and selective impression techniques demonstrated greater tissue displacement compared with the functional reline method, with the selective pressure technique showing the most favorable overall performance for distal extension removable partial dentures. These findings suggest that controlled functional recording of the edentulous ridge may promote favorable tissue recording, defined as capturing the supporting mucosa in a physiologically acceptable and controlled functional state. Such recording facilitates more balanced load distribution between the supporting mucosa and abutment teeth, thereby potentially enhancing denture stability.

## Introduction

Rehabilitation of partially edentulous patients with bilateral distal extension removable partial dentures (RPDs) presents a significant biomechanical challenge in prosthodontic practice [1,2]. In Kennedy Class I situations, the absence of posterior abutment support results in a prosthesis that is supported anteriorly by abutment teeth and posteriorly by the edentulous ridge [3]. Unlike tooth-supported prostheses, distal extension RPDs derive support from two biologically distinct structures: the relatively rigid periodontal ligament-supported abutment teeth and the compressible mucosa covering the residual ridge [4]. This disparity in resiliency between the two supporting structures creates complex biomechanical interactions during function [5].

Under occlusal loading, abutment teeth exhibit minimal displacement because of the viscoelastic properties of the periodontal ligament, whereas the edentulous ridge mucosa demonstrates significantly greater vertical displacement [6]. This difference may lead to rotation of the denture base around a fulcrum line passing through the terminal abutments [1,7]. Such rotational movements can generate torque and lateral forces on abutment teeth, potentially resulting in clasp fatigue, tooth mobility, periodontal breakdown, and progressive residual ridge resorption [4,8]. Therefore, achieving biomechanical harmony between tooth and tissue support is fundamental to the long-term success of distal extension RPDs [2,5].

The soft tissues underlying the distal extension denture base serve both mechanical and physiological roles [9]. Mechanically, they function as a stress-distributing medium, transmitting masticatory loads to the underlying bone [6]. Physiologically, the vascular supply of the mucosa contributes to the maintenance of bone vitality and tissue health [10]. Excessive or uneven loading due to an ill-fitting denture base may impair circulation, accelerate ridge resorption, and compromise prosthesis stability [9,10]. Consequently, the impression procedure assumes critical importance, as it determines how the supporting tissues are recorded and how functional forces will subsequently be distributed [3,7].

Various impression philosophies have been proposed to manage the resiliency differences between teeth and mucosa in distal extension cases, including mucostatic, mucocompressive, and selective pressure concepts [3,7]. Among the clinically employed techniques, the Hindels method, the selective pressure technique, and the functional reline method represent distinct approaches to recording supporting tissues under varying degrees of pressure [1,3,7].

In recent years, advances in prosthodontic research have further emphasized the biomechanical challenges associated with distal extension removable partial dentures (RPDs). Contemporary studies have highlighted the importance of impression philosophy in controlling denture base movement, optimizing stress distribution between abutment teeth and the supporting mucosa, and improving prosthesis stability during function. Recent investigations employing digital analysis and finite element modeling have demonstrated that impression techniques capable of producing controlled functional displacement of the residual ridge may enhance load distribution and reduce unfavorable stresses on supporting structures in distal extension situations [11,12]. Therefore, continued evaluation of impression procedures remains important to refine clinical protocols and improve treatment outcomes in distal extension RPD therapy.

However, quantitative evidence directly comparing the amount of vertical tissue displacement produced by these methods in bilateral distal extension cases remains limited [8-10].

Therefore, the present study was undertaken to evaluate and compare the vertical tissue displacement produced by three impression techniques: the Hindels method, the selective pressure method, and the functional reline method in bilateral distal extension removable partial denture cases. In this study, tissue displacement refers to the dimensional differences measured on the definitive casts obtained from each impression technique, representing the extent of mucosal displacement that occurred during impression making. The null hypothesis of this study was that there would be no significant difference in tissue displacement among the Hindels impression method, the selective pressure method, and the functional reline method used for recording bilateral distal extension ridges.

## Materials and methods

This in vivo comparative experimental study was conducted to evaluate vertical tissue displacement produced by three impression techniques used in bilateral distal extension removable partial dentures (RPDs), namely, the Hindels impression method, the selective pressure method, and the functional reline method. The study was carried out in the Department of Prosthodontics and Crown & Bridge at Chandra Dental College and Hospital, Barabanki, in collaboration with Panna Dental Laboratory, Lucknow.

The study protocol was approved by the Ethical Committee, Chandra Dental College and Hospital (CDCH/EC/PROSTH/2023-26/018). Written informed consent was obtained from the patient before participation, and the study was conducted in accordance with the principles of the Declaration of Helsinki.

**Figure 1:**
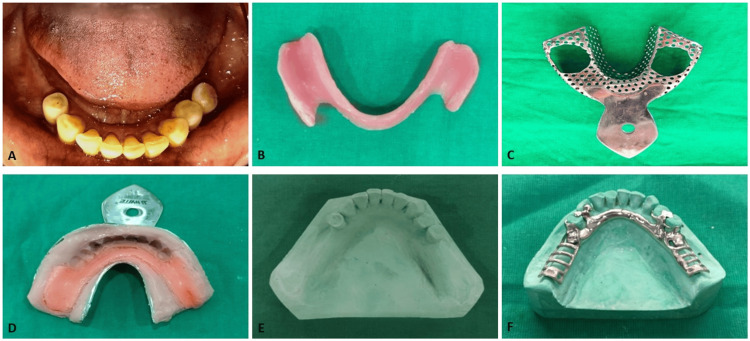
Hindels impression technique for mandibular distal extension removable partial denture A: Intraoral view of partially edentulous mandibular arch; B: Special tray for the Hindels method; C: Stock metal tray for the Hindels method; D: Final impression using the Hindels method; E: Definitive cast obtained using the Hindels method (group A sample); F: Cast partial denture framework

A 42-year-old female patient with bilaterally missing mandibular posterior teeth consistent with Kennedy Class I classification was selected for the study (Figure 1A). The patient was selected based on convenience from patients reporting to the department who fulfilled the predefined inclusion and exclusion criteria. The patient was systemically healthy and exhibited no evidence of local oral pathology. The residual ridges were well-formed with adequate ridge height and firm mucosal covering, without signs of flabby or hypermobile tissue. Inclusion criteria included the presence of bilateral distal extension edentulous areas in the mandible, well-formed residual ridges, and the absence of systemic disease. Exclusion criteria comprised medically compromised status, restricted mouth opening, flabby or hypermobile residual ridges, and unwillingness to participate in the study. Oral prophylaxis was performed before initiation of the impression procedures to ensure optimal periodontal and mucosal health.

A total of 15 definitive casts were fabricated from the same patient and allocated into three groups, with five casts in each group. This approach was adopted to eliminate interpatient variability in ridge morphology, mucosal resiliency, and neuromuscular factors, thereby allowing a standardized comparison of the impression procedures. All impressions were made sequentially during the same clinical session, with adequate intervals provided between impressions to allow recovery of the mucosal tissues and minimize the influence of residual tissue deformation. Group A consisted of casts obtained using the Hindels impression technique, group B included casts fabricated using the selective pressure impression technique, and group C comprised casts obtained using the functional reline technique.

For group A, a preliminary impression was made using irreversible hydrocolloid in a stock tray to obtain a primary cast. A custom acrylic tray was fabricated on the primary cast and selectively relieved by approximately 1 mm over the ridge crest, while the buccal shelf region was left unrelieved to function as the primary stress-bearing area (Figure 1B). A zinc oxide eugenol impression was then made to record the tissues in a passive, minimally compressed state. To simulate functional loading and establish the relationship between abutment teeth and supporting mucosa, a perforated metal tray was used to make a secondary impression with alginate (Figure 1C). Controlled digital pressure was applied through tray openings over the distal extension area during impression making (Figure 1D) by the same operator to ensure consistency. Pressure was standardized by applying manual pressure with the operator’s index finger until initial mucosal resistance was perceived. All procedures were performed by the same operator to minimize variability. Measurements were repeated to verify consistency, and the mean value was used for statistical analysis. The impression assembly was removed as a single unit and poured in type III dental stone to obtain the definitive cast (Figure 1E), and a cast partial denture framework was fabricated on the cast (Figure 1F). This procedure was repeated to fabricate five casts for each technique to allow repeated measurements and improve the reliability of the comparative analysis.

**Figure 2:**
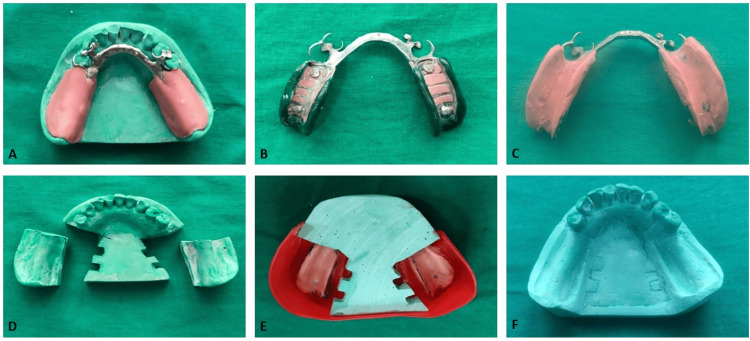
Selective pressure impression technique for mandibular distal extension removable partial denture A: Special tray fabricated for selective pressure method; B: Border molding; C: Final impression using selective pressure method; D: Edentulous areas cut in the anatomical master cast and vertical grooves prepared on the cut walls of cast; E: Beading and boxing of the impression obtained by selective pressure method; F: Master cast obtained using altered cast technique (group B sample)

For group B, an anatomic impression was obtained using irreversible hydrocolloid to fabricate a master cast on which a metal partial denture framework was constructed. A closely adapted custom tray was fabricated over the distal extension ridge area without the use of a spacer (Figure 2A). Approximately 1 mm of relief was provided over the ridge crest, while the buccal shelf region was minimally relieved to enhance support. Border molding was carried out using low-fusing modeling compound to capture functional vestibular contours (Figure 2B). A final impression was made using zinc oxide eugenol paste under controlled pressure conditions (Figure 2C). The altered cast technique was then employed by sectioning the edentulous ridge portion of the master cast (Figure 2D) and re-pouring the functional impression in continuity (Figure 2E) with the remaining cast to obtain a corrected ridge form (Figure 2F). Five definitive casts were produced using this protocol.

**Figure 3:**
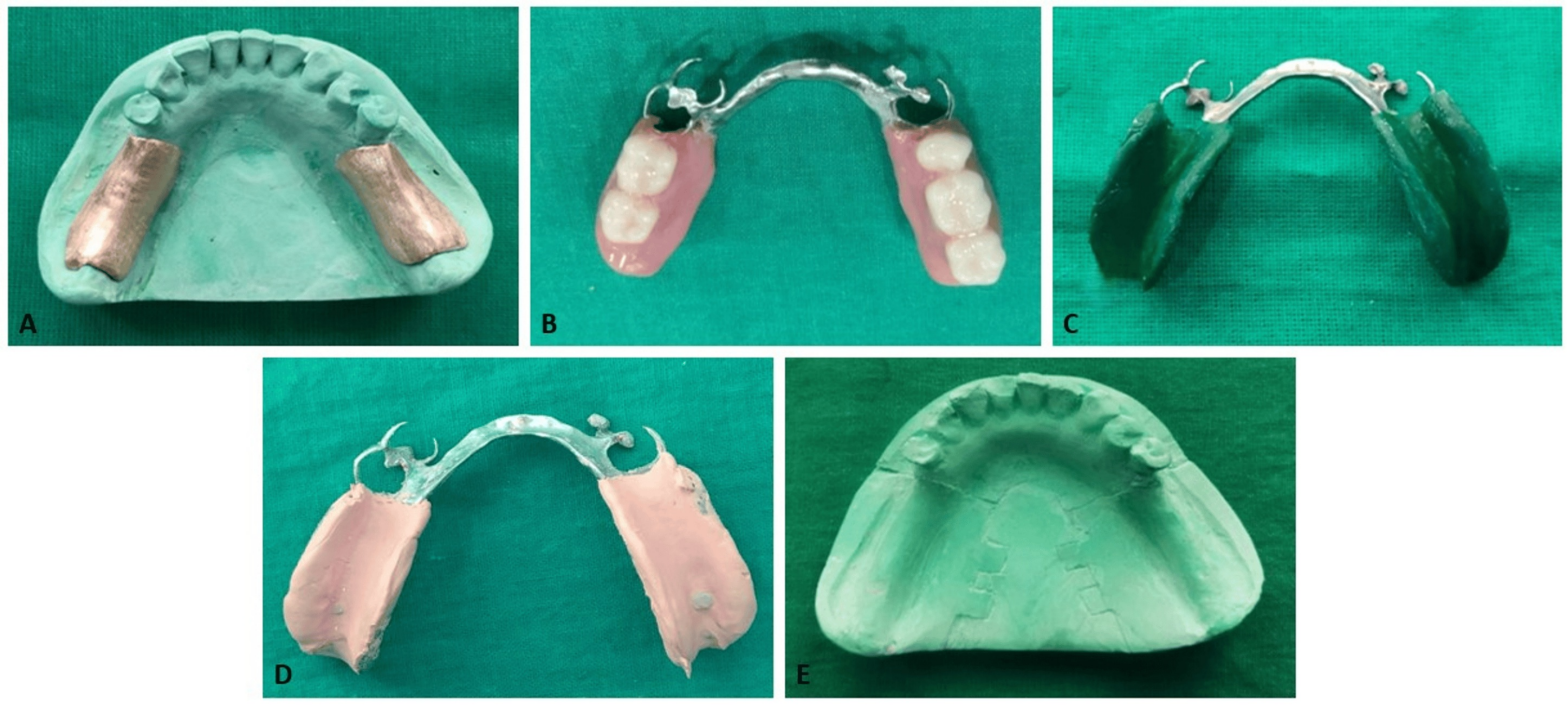
Functional reline impression technique for mandibular distal extension removable partial denture A: Metal spacer; B: Processed cast partial denture; C: Impression using low-fusing impression compound; D: Final impression using functional reline method; E: Master cast obtained using altered cast technique (group C sample)

For group C, an anatomic impression was made and a framework fabricated in a similar manner. A metal spacer was adapted over the ridge prior to processing the denture base to create uniform relief beneath the intaglio surface (Figure 3A). After processing the cast partial denture, the spacer was removed to provide space for impression material (Figure 3B). Low-fusing modeling compound was applied to the tissue surface of the denture base, tempered, and inserted intraorally (Figure 3C). Functional movements were guided to shape the compound under dynamic conditions. Approximately 1 mm of compound was subsequently removed to create space for a wash impression using zinc oxide eugenol paste (Figure 3D). Subsequently, a thin corrective wash of zinc oxide eugenol impression material was applied to refine the surface detail of the impression. Care was taken to maintain a minimal thickness of the wash layer so that the underlying compound provided the primary functional support during impression-making. The completed impression was evaluated for accuracy and poured using the altered cast technique (Figure 3E). Five casts were fabricated using this method.

**Figure 4:**
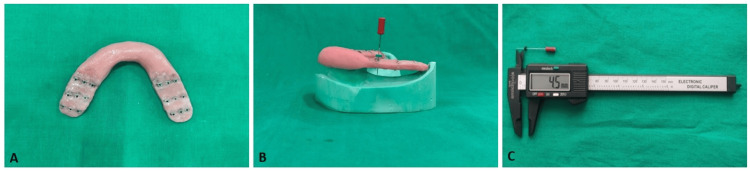
Method for evaluation of vertical tissue displacement A: Autopolymerising acrylic resin platform; B: Endodontic K file inserted through the buccal tube; C: Digital vernier caliper for measuring tissue displacement

To evaluate vertical tissue displacement, a standardized acrylic reference platform was fabricated at the level of the occlusal surfaces of the remaining teeth and extended bilaterally over the distal extension areas (Figure 4A). Orthodontic buccal tubes were positioned in three standardized reference regions corresponding to the anterior, middle, and posterior portions of the edentulous ridge on each side. The distance from the distal surface of the terminal abutment tooth to the retromolar pad was measured and divided into three equal segments to define these regions. Buccal tubes were placed at the midpoint of each segment, and a transparent template fabricated from the initial cast was used as a positioning guide to ensure consistent placement of the buccal tubes across all casts. A size 25 endodontic K-file was inserted through each buccal tube until it contacted the ridge surface, and a rubber stopper was adjusted at the level of the tube (Figure 4B). The distance between the file tip and the rubber stopper was measured using a calibrated digital vernier caliper with an accuracy of 0.01 mm (Figure 4C). The reference platform was sequentially positioned on each of the fifteen casts, and measurements were recorded. To ensure measurement reliability, the land areas of all casts were standardized during trimming to maintain consistent base height and orientation. The acrylic reference platform was fabricated from the initial reference cast and designed to sit on reproducible landmarks, allowing consistent positioning relative to the edentulous saddle on all casts. The measured distances, expressed in millimeters, represented vertical tissue displacement produced by each impression technique.

Data were expressed as mean ± standard deviation. Normality of distribution was assessed using the Shapiro-Wilk test, and homogeneity of variance was evaluated using Levene’s test. Inter-group comparisons were performed using one-way analysis of variance (ANOVA), and pairwise comparisons were conducted using Tukey’s honestly significant difference post hoc test. A two-tailed p value of less than 0.05 was considered statistically significant. Statistical analysis was performed using IBM SPSS Statistics, Version 22.0 (IBM Corp., Armonk, NY, USA).

## Results

Intra-group comparison

The tissue displacement at the anterior, middle, and posterior regions of the distal extension ridge using the Hindels impression method is presented (Table 1). The mean (± standard deviation) tissue displacement recorded at the anterior, middle, and posterior regions was 17.46 ± 0.03 mm, 17.49 ± 0.07 mm, and 17.52 ± 0.05 mm, respectively. The highest mean tissue displacement was observed in the posterior region, followed by the middle region, while the anterior region demonstrated the lowest mean value (anterior < middle < posterior). Measurements were obtained using a digital vernier caliper with high precision. To ensure reliability, each measurement was repeated, and the mean value was recorded for analysis. 

**Table 1:** Comparison of mean tissue displacement (mm) at the anterior, middle, and posterior regions of the edentulous ridge using the Hindels method Values are presented as mean ± standard deviation (SD) for each region (n = 5 per group). Inter-regional comparison was performed using one-way analysis of variance (ANOVA), and the corresponding F and P values are reported. A P value < 0.05 was considered statistically significant.

Region	n	Tissue displacement (mm) (Mean ± SD)	F value	P value
Anterior	5	17.46 ± 0.03	1.90	0.191
Middle	5	17.49 ± 0.07		
Posterior	5	17.52 ± 0.05		

One-way analysis of variance (ANOVA) was performed to compare mean tissue displacement among the three regions. The analysis revealed no statistically significant difference in tissue displacement (F = 1.90, P = 0.191), indicating comparable tissue displacement across the anterior, middle, and posterior regions.

Post hoc pairwise comparisons using Tukey’s honestly significant difference test further confirmed the absence of statistically significant differences between any two regions (Table 2). The comparison between the anterior and middle regions showed a mean difference of 0.02 mm (q = 1.12, P > 0.05). Similarly, the anterior and posterior regions demonstrated a mean difference of 0.06 mm (q = 2.74, P > 0.05), and the middle and posterior regions showed a mean difference of 0.04 mm (q = 1.63, P > 0.05).

**Table 2:** Pairwise comparison of mean differences in tissue displacement (mm) among the anterior, middle, and posterior regions using the Hindels impression method assessed by Tukey’s post hoc test Statistical analysis was performed using Tukey’s post hoc test following one-way ANOVA. The table presents the mean difference, Tukey q statistic, corresponding P value, and 95% confidence interval (CI) for each inter-regional comparison. A P value < 0.05 was considered statistically significant. diff., difference; CI, confidence interval; q value, Tukey test statistic

Comparison between regions	Mean diff.	q value	P value	95% CI of diff.
Anterior vs. middle	0.03	1.12	P > 0.05	0.062 to 0.114
Anterior vs. posterior	0.06	2.74	P > 0.05	0.024 to 0.152
Middle vs. posterior	0.04	1.63	P > 0.05	0.050 to 0.126

Overall, no statistically significant difference in tissue displacement was observed among the three regions when Hindels impression method was employed (P > 0.05).

The tissue displacement at the anterior, middle, and posterior regions of the distal extension ridge using the selective pressure method is presented in Table 3. The mean (± standard deviation) tissue displacement recorded at the anterior, middle, and posterior regions was 17.52 ± 0.03 mm, 17.55 ± 0.09 mm, and 17.61 ± 0.05 mm, respectively. The posterior region demonstrated the highest mean tissue displacement, followed by the middle region, while the anterior region exhibited the lowest mean value (anterior < middle < posterior).

**Table 3:** Comparison of mean tissue displacement (mm) at the anterior, middle, and posterior regions of the ridge using the selective pressure impression technique Values are expressed as mean ± standard deviation (SD) for each region (n = 5). Inter-regional differences were analyzed using one-way analysis of variance (ANOVA), and the corresponding F and P values are presented. A P value < 0.05 was considered statistically significant. No statistically significant difference was observed among the three regions (P = 0.116).

Region	n	Tissue displacement (mm) (Mean ± SD)	F value	P value
Anterior	5	17.52 ± 0.03	2.59	0.116
Middle	5	17.55 ± 0.09		
Posterior	5	17.61 ± 0.05		

One-way analysis of variance (ANOVA) was performed to compare mean tissue displacement among the three regions. The analysis revealed no statistically significant difference in tissue displacement across the regions (F = 2.59, P = 0.116), indicating comparable tissue recording within the anterior, middle, and posterior areas.

Post hoc pairwise comparisons using Tukey’s honestly significant difference test further demonstrated no statistically significant differences between any two regions (Table 4). The comparison between the anterior and middle regions showed a mean difference of 0.03 mm (q = 1.08, P > 0.05). The anterior and posterior regions exhibited a mean difference of 0.09 mm (q = 3.17, P > 0.05), while the middle and posterior regions showed a mean difference of 0.06 mm (q = 2.09, P > 0.05).

**Table 4:** Pairwise comparison of mean differences in tissue displacement (mm) among the anterior, middle, and posterior regions using the selective pressure impression technique Inter-regional comparisons were performed using Tukey’s post hoc test following one-way ANOVA. The table presents the mean difference, Tukey q statistic, corresponding P value, and 95% confidence interval (CI) for each comparison. No statistically significant differences were observed between the regions (P > 0.05). diff., difference; CI, confidence interval; q value, Tukey test statistic

Comparison between regions	Mean diff.	q value	P value	95% CI of diff.
Anterior vs. middle	0.03	1.08	P > 0.05	0.075 to 0.135
Anterior vs. posterior	0.09	3.17	P > 0.05	0.017 to 0.193
Middle vs. posterior	0.06	2.09	P > 0.05	0.047 to 0.163

Overall, no statistically significant difference in tissue displacement was observed among the three regions when the selective pressure method was used (P > 0.05).

The tissue displacement at the anterior, middle, and posterior regions of the distal extension ridge using the functional reline method is presented (Table 5). The mean (± standard deviation) tissue displacement recorded at the anterior, middle, and posterior regions was 15.59 ± 0.09 mm, 15.63 ± 0.05 mm, and 15.68 ± 0.06 mm, respectively. The posterior region demonstrated the highest mean tissue displacement, followed by the middle region, while the anterior region exhibited the lowest mean value (anterior < middle < posterior).

**Table 5:** Comparison of mean tissue displacement (mm) at the anterior, middle, and posterior regions of the ridge using the functional reline impression technique Values are expressed as mean ± standard deviation (SD) for each region (n = 5). Inter-regional differences were analyzed using one-way analysis of variance (ANOVA), and the corresponding F and P values are presented. A P value < 0.05 was considered statistically significant. No statistically significant difference was observed among the three regions (P = 0.158).

Region	n	Tissue displacement (mm) (Mean ± SD)	F value	P value
Anterior	5	15.59 ± 0.09	2.16	0.158
Middle	5	15.63 ± 0.05		
Posterior	5	15.68 ± 0.06		

One-way analysis of variance (ANOVA) was performed to compare mean tissue displacement among the three regions. The analysis revealed no statistically significant difference in tissue displacement across the regions (F = 2.16, P = 0.158), indicating comparable tissue recording within the anterior, middle, and posterior regions.

Post hoc pairwise comparisons using Tukey’s honestly significant difference test further demonstrated no statistically significant differences between any two regions (Table 6). The comparison between the anterior and middle regions showed a mean difference of 0.03 mm (q = 1.10, P > 0.05). The anterior and posterior regions exhibited a mean difference of 0.09 mm (q = 2.91, P > 0.05), while the middle and posterior regions showed a mean difference of 0.06 mm (q = 1.81, P > 0.05).

**Table 6:** Pairwise comparison of mean differences in tissue displacement (mm) among the anterior, middle, and posterior regions using the functional reline method assessed by Tukey’s post hoc test Inter-regional comparisons were performed using Tukey’s post hoc test following one-way ANOVA. The table presents the mean difference, Tukey q statistic, corresponding P value, and 95% confidence interval (CI) for each comparison. No statistically significant differences were observed between any of the regional comparisons (P > 0.05). diff., difference; CI, confidence interval; q value, Tukey test statistic

Comparison between regions	Mean diff.	q value	P value	95% CI of diff.
Anterior vs. middle	0.03	1.10	P > 0.05	0.083 to 0.151
Anterior vs. posterior	0.09	2.91	P > 0.05	0.027 to 0.207
Middle vs. posterior	0.06	1.81	P > 0.05	0.061 to 0.173

Overall, no statistically significant difference in tissue displacement was observed among the three regions when the functional reline method was employed (P > 0.05).

Inter-group comparison

The comparison of tissue displacement at the anterior region among the three impression techniques (the Hindels impression method, selective pressure method, and functional reline method) is presented (Table 7). The mean (± standard deviation) tissue displacement recorded using the Hindels impression method, selective pressure method, and functional reline method was 17.46 ± 0.03 mm, 17.52 ± 0.03 mm, and 15.59 ± 0.09 mm, respectively. The selective pressure method demonstrated the highest mean tissue displacement, followed closely by the Hindels impression method, while the functional reline method showed the lowest mean value (functional reline < Hindels < selective pressure).

**Table 7:** Comparison of mean tissue displacement (mm) at the anterior region using three different impression techniques Hindels method, selective pressure method, and functional reline method. Values are expressed as mean ± standard deviation (SD) (n = 5 per group). Intergroup comparison was performed using one-way analysis of variance (ANOVA), and the corresponding F and P values are reported. A statistically significant difference was observed among the three methods (P < 0.001).

Method	n	Tissue displacement (mm) (Mean ± SD)	F value	P value
Hindels method	5	17.46 ± 0.03	1713.00	< 0.001
Selective pressure method	5	17.52 ± 0.03		
Functional reline method	5	15.59 ± 0.09		

One-way analysis of variance (ANOVA) revealed a statistically significant difference in mean tissue displacement among the three impression techniques (F = 1713.00, P < 0.001), indicating that the method of impression significantly influenced tissue displacement at the anterior region.

Post hoc pairwise comparisons using Tukey’s honestly significant difference test demonstrated no statistically significant difference between the Hindels impression method and the selective pressure method (mean difference = 0.06 mm; q = 2.27; P > 0.05) (Table 8). However, both the Hindels impression method and the selective pressure method exhibited significantly greater tissue displacement compared to the functional reline method. The comparison between the Hindels and functional reline methods showed a mean difference of 1.87 mm (q = 70.54; P < 0.001), while the comparison between the selective pressure and functional reline methods demonstrated a mean difference of 1.93 mm (q = 72.80; P < 0.001) (Table 8).

**Table 8:** Pairwise comparison of tissue displacement (mm) at the anterior region among the three impression techniques using Tukey’s post hoc test The table presents the mean difference, Tukey q statistic, corresponding P value, and 95% confidence interval (CI) for each inter-method comparison. A statistically significant difference was observed between the functional reline method and both the Hindels and selective pressure methods (P < 0.001), whereas no significant difference was found between the Hindels and selective pressure methods (P > 0.05). diff., difference; CI, confidence interval; q value, Tukey test statistic

Comparison between methods	Mean diff.	q value	P value	95% CI of diff.
Hindels vs. selective pressure	0.06	2.27	P > 0.05	0.040 to 0.160
Hindels vs. functional reline	1.87	70.54	P < 0.001	1.768 to 1.968
Selective pressure vs. functional reline	1.93	72.80	P < 0.001	1.828 to 2.028

Overall, tissue displacement at the anterior region was significantly higher with both the Hindels and selective pressure methods compared to the functional reline method (P < 0.001), whereas no statistically significant difference was observed between Hindels and selective pressure methods (P > 0.05).

The comparison of tissue displacement at the middle region among the three impression techniques (the Hindels method, selective pressure method, and functional reline method) is presented (Table 9). The mean (± standard deviation) tissue displacement recorded using the Hindels, selective pressure, and functional reline methods was 17.49 ± 0.07 mm, 17.55 ± 0.09 mm, and 15.63 ± 0.05 mm, respectively. The selective pressure method demonstrated the highest mean tissue displacement, followed by the Hindels method, while the functional reline method exhibited the lowest mean value (functional reline < Hindels < selective pressure).

**Table 9:** Comparison of mean tissue displacement (mm) at the middle region among the three impression techniques Hindels method, selective pressure method, and functional reline method. Values are presented as mean ± standard deviation (SD) (n = 5 per group). Intergroup comparison was performed using one-way analysis of variance (ANOVA), and the corresponding F and P values are reported. A statistically significant difference was observed among the three impression methods in the middle region (P < 0.001).

Method	n	Tissue displacement (mm) (Mean ± SD)	F value	P value
Hindles method	5	17.49 ± 0.07	1283.00	< 0.001
Selective pressure method	5	17.55 ± 0.09		
Functional reline method	5	15.63 ± 0.05		

One-way analysis of variance (ANOVA) indicated a statistically significant difference in tissue displacement among the three methods at the middle region (P < 0.001).

Post hoc pairwise comparisons using Tukey’s honestly significant difference test demonstrated no statistically significant difference between the Hindels and selective pressure methods (mean difference = 0.06 mm; q = 2.10; P > 0.05) (Table 10). However, both the Hindels and the selective pressure methods showed significantly greater tissue displacement compared to the functional reline method. The comparison between the Hindels and functional reline methods revealed a mean difference of 1.86 mm (q = 60.97; P < 0.001), while the selective pressure and functional reline methods demonstrated a mean difference of 1.92 mm (q = 63.07; P < 0.001) (Table 10).

**Table 10:** Pairwise comparison of mean differences in tissue displacement (mm) among the three impression techniques at the middle region using Tukey’s post hoc test The table presents the mean difference, Tukey q statistic, corresponding P value, and 95% confidence interval (CI) for each inter-method comparison. A statistically significant difference was observed between the functional reline method and both Hindels and selective pressure methods (P < 0.001), whereas no significant difference was found between Hindels and selective pressure methods (P > 0.05). diff., difference; CI, confidence interval; q value, Tukey test statistic

Comparison between methods	Mean diff.	q value	P value	95% CI of diff.
Hindels vs. selective pressure	0.06	2.10	P > 0.05	0.051 to 0.179
Hindels vs. functional reline	1.86	60.97	P < 0.001	1.745 to 1.975
Selective pressure vs. functional reline	1.92	63.07	P < 0.001	1.809 to 2.039

Overall, tissue displacement at the middle region was significantly higher with both the Hindels and selective pressure methods compared to the functional reline method (P < 0.001), whereas no statistically significant difference was observed between Hindels and selective pressure methods (P > 0.05).

The comparison of tissue displacement at the posterior region among the three impression techniques (the Hindels method, selective pressure method, and functional reline method) is presented (Table 11). The mean (± standard deviation) tissue displacement recorded using the Hindels method, selective pressure method, and functional reline method was 17.52 ± 0.05 mm, 17.61 ± 0.05 mm, and 15.68 ± 0.06 mm, respectively. The selective pressure method demonstrated the highest mean tissue displacement, followed by the Hindels method, while the functional reline method exhibited the lowest mean value (functional reline < Hindels < selective pressure).

**Table 11:** Comparison of mean tissue displacement (mm) at the posterior region obtained using three different impression techniques Values are expressed as mean ± standard deviation (SD) (n = 5 per group). Intergroup comparison was performed using one-way analysis of variance (ANOVA), and the corresponding F and P values are reported. A statistically significant difference was observed among the three impression methods at the posterior region (P < 0.001).

Method	n	Tissue displacement (mm) (Mean ± SD)	F value	P value
Hindels method	5	17.52 ± 0.05	1857.00	< 0.001
Selective pressure method	5	17.61 ± 0.05		
Functional reline method	5	15.68 ± 0.06		

One-way analysis of variance (ANOVA) revealed a statistically significant difference in tissue displacement among the three impression techniques at the posterior region (F = 1857.00, P < 0.001), indicating that the choice of impression method significantly influenced tissue displacement.

Post hoc pairwise comparisons using Tukey’s honestly significant difference test demonstrated no statistically significant difference between the Hindels and selective pressure methods (mean difference = 0.08 mm; q = 3.33; P > 0.05) (Table 12). However, both the Hindels and the selective pressure methods showed significantly greater tissue displacement compared to the functional reline method. The comparison between the Hindels and functional reline methods revealed a mean difference of 1.84 mm (q = 72.93; P < 0.001), while the selective pressure and functional reline methods demonstrated a mean difference of 1.92 mm (q = 76.25; P < 0.001) (Table 12).

**Table 12:** Pairwise comparison of mean differences in tissue displacement (mm) at the posterior region among the three impression techniques using Tukey’s post hoc test The table presents the mean difference, Tukey q statistic, corresponding P value, and 95% confidence interval (CI) for each inter-method comparison. A statistically significant difference was observed between the functional reline method and both the Hindels and selective pressure methods (P < 0.001), whereas no significant difference was found between the Hindels and selective pressure methods (P > 0.05). diff., difference; CI, confidence interval; q value, Tukey test statistic

Comparison between methods	Mean diff.	q value	P value	95% CI of diff.
Hindels vs. selective pressure	0.08	3.33	P > 0.05	0.011 to 0.179
Hindels vs. functional reline	1.84	72.93	P < 0.001	1.747 to 1.937
Selective pressure vs. functional reline	1.93	76.25	P < 0.001	1.831 to 2.021

Overall, tissue displacement at the posterior region was significantly higher with both Hindels and selective pressure methods compared to the functional reline method (P < 0.001), whereas no statistically significant difference was observed between Hindels and selective pressure methods (P > 0.05).

Overall comparison

The overall tissue displacement, calculated as the average of the anterior, middle, and posterior regions, for the three impression techniques (the Hindels method, selective pressure method, and functional reline method) is presented (Table 13). The overall mean (± standard deviation) tissue displacement recorded using the Hindels method, selective pressure method, and functional reline method was 17.49 ± 0.05 mm, 17.56 ± 0.05 mm, and 15.63 ± 0.04 mm, respectively. The selective pressure method demonstrated the highest overall mean tissue displacement, followed by the Hindels method, while the functional reline method exhibited the lowest mean value (functional reline < Hindels < selective pressure).

**Table 13:** Comparison of overall mean tissue displacement (mm) among the three impression techniques Values are expressed as mean ± standard deviation (SD) (n = 5 per group). Intergroup comparison was performed using one-way analysis of variance (ANOVA), and the corresponding F and P values are presented. A statistically significant difference was observed among the three impression methods in overall tissue displacement (P < 0.001).

Method	n	Tissue displacement (mm) (Mean ± SD)	F value	P value
Hindels method	5	17.49 ± 0.05	3005.00	< 0.001
Selective pressure method	5	17.56 ± 0.05		
Functional reline method	5	15.63 ± 0.04		

One-way analysis of variance (ANOVA) revealed a statistically significant difference in overall tissue displacement among the three impression techniques (F = 3005.00, P < 0.001), indicating that the impression method significantly influenced the extent of tissue displacement.

Post hoc pairwise comparisons using Tukey’s honestly significant difference test demonstrated no statistically significant difference between the Hindels and selective pressure methods (mean difference = 0.07 mm; q = 3.41; P > 0.05) (Table 14). However, both the Hindels and selective pressure methods showed significantly greater overall tissue displacement compared to the functional reline method. The comparison between the Hindels and functional reline methods revealed a mean difference of 1.86 mm (q = 93.19; P < 0.001), while the selective pressure and functional reline methods demonstrated a mean difference of 1.92 mm (q = 96.60; P < 0.001) (Table 14).

**Table 14:** Pairwise comparison of differences in overall mean tissue displacement (mm) among the three impression techniques using Tukey’s post hoc test The table presents the mean difference, Tukey q statistic, corresponding P value, and 95% confidence interval (CI) for each inter-method comparison. A statistically significant difference was observed between the functional reline method and both the Hindels and selective pressure methods (P < 0.001), whereas no significant difference was found between the Hindels and selective pressure methods (P > 0.05). diff., difference; CI, confidence interval; q value, Tukey test statistic

Comparison between methods	Mean diff.	q value	P value	95% CI of diff.
Hindels vs. selective pressure	0.07	3.41	P > 0.05	0.007 to 0.143
Hindels vs. functional reline	1.86	93.19	P < 0.001	1.781 to 1.931
Selective pressure vs. functional reline	1.92	96.60	P < 0.001	1.849 to 1.999

Overall, the selective pressure method produced the highest mean tissue displacement, followed closely by the Hindels method, whereas the functional reline method demonstrated the least tissue displacement. Both the Hindels and selective pressure methods showed significantly greater overall tissue displacement compared to the functional reline method (P < 0.001), while no statistically significant difference was observed between the Hindels and selective pressure methods (P > 0.05). Additionally, the overall mean tissue displacement achieved using the selective pressure method was approximately 0.4% higher than that of the Hindels method and approximately 11.0% higher than that of the functional reline method.

## Discussion

The management of distal-extension removable partial dentures (RPDs) presents a biomechanical challenge because support is derived from two biologically dissimilar structures: the relatively rigid abutment teeth and the compressible mucosa covering the edentulous ridge [1,2]. Retention is achieved through clasps or precision attachments connected to the abutment teeth [3]. Under vertical masticatory loading, abutment teeth exhibit minimal displacement due to the viscoelastic properties of the periodontal ligament, whereas the distal extension mucosa demonstrates greater compressibility [9]. This disparity in resiliency promotes rotation of the denture base around a fulcrum line passing through the terminal abutment rests, increasing posterior tissue loading and potentially accelerating residual ridge resorption [10]. Additionally, unfavorable stress transfer may compromise the periodontal integrity of abutment teeth over time.

Functional impression techniques have been advocated to harmonize support between resilient mucosa and relatively non-resilient abutment teeth. These approaches aim to record the edentulous ridge under simulated functional loading conditions and relate the displaced ridge form to the remaining dentition, thereby minimizing denture base movement and improving stress distribution.

Although intra-group differences in tissue displacement among anterior, middle, and posterior regions were not statistically significant in the present investigation, a consistent pattern of greater displacement in the posterior region was observed. This trend aligns with previous findings reported by Vahidi [13] and Madihalli et al., who demonstrated increased compressibility and functional displacement in posterior ridge tissues, particularly in the retromolar pad region [7]. Similar observations were reported by Aljudy, who found greater posterior tissue displacement when different impression and border-molding materials were used in altered cast procedures [14]. Collectively, these studies support the concept of a resiliency gradient along the residual ridge.

The influence of impression technique on denture movement has been well documented. Holmes demonstrated that denture displacement under occlusal loading is affected by the impression material and procedure, with altered cast techniques producing reduced movement at insertion [15]. Leupold et al. reported greater distortion of mobile mucosa with stock tray impressions compared to altered cast techniques, and subsequent investigations confirmed that altered cast procedures resulted in minimal vertical denture base movement in distal extension cases [16]. These findings emphasize the importance of controlled impression philosophies in optimizing prosthesis stability.

In the present study, the selective pressure technique exhibited the highest overall tissue displacement, followed closely by the Hindels method, whereas the functional reline method demonstrated the least displacement. These results are consistent with those reported by Madihalli et al., who observed superior tissue displacement with the selective pressure technique compared to the Hindels and functional reline methods [7]. The selective pressure approach permits controlled loading of primary stress-bearing areas while relieving sensitive regions, and through the altered cast procedure, records the ridge in a functionally displaced yet physiologically acceptable form. The Hindels method also facilitates functional recording; however, slightly lower displacement values were noted, although without statistical significance, indicating comparable biomechanical performance.

Conversely, the functional reline method demonstrated reduced and less controlled tissue displacement. The use of low-fusing compounds may lead to indiscriminate compression of both resilient and delicate mucosa, resulting in uneven displacement patterns and potentially less favorable stress distribution during function.

Overall, the findings of this study underscore that impression technique significantly influences tissue displacement and support distribution between abutment teeth and the residual ridge in distal extension situations [1,2]. Techniques that provide controlled functional displacement appear to enhance biomechanical balance and prosthesis stability.

In interpreting these findings, it is also important to consider potential sources of measurement variability. Although standardized procedures were employed during impression making and cast measurements to minimize operator-related variation, minor differences in pressure application, positioning of measurement references, or mucosal recovery between procedures may influence the magnitude of recorded tissue displacement. Nevertheless, repeated measurements and consistent methodology were used to enhance reliability. Therefore, the observed differences should be interpreted in the context of these methodological considerations, recognizing that small variations in measured displacement may not necessarily translate into proportionally large clinical effects.

Although the present study evaluated tissue displacement on casts rather than direct clinical outcomes, the findings have relevant clinical implications. Controlled tissue displacement during impression making is critical in distal extension removable partial dentures because excessive denture base movement can contribute to patient discomfort, instability during mastication, accelerated residual ridge resorption, and increased stress on abutment teeth. Impression techniques that achieve more physiologic and controlled mucosal displacement may therefore improve denture stability and load distribution, potentially enhancing patient comfort and long-term prosthesis performance. However, as the present investigation assessed displacement only at the cast level, further longitudinal clinical studies evaluating patient-reported outcomes, ridge preservation, and prosthesis survival are necessary to confirm the clinical benefits of these impression techniques.

The limitations of this study include its single-patient design and limited sample size, which restrict generalizability. Variations in ridge morphology and mucosal characteristics may influence displacement patterns. In addition, repeated impression procedures performed on the same residual ridge may temporarily alter mucosal compressibility and potentially influence tissue displacement measurements. Furthermore, the evaluated techniques are operator-dependent and technique-sensitive, which may introduce procedural variability. Future research incorporating larger cohorts, objective digital measurement methods, and longitudinal clinical follow-up is warranted to further elucidate the long-term biomechanical implications of different impression philosophies.

## Conclusions

Variations in tissue displacement were observed across different regions of the edentulous ridge, with relatively greater displacement noted in the posterior area compared with the middle and anterior regions. These findings underscore the influence of anatomical differences in residual ridge tissues on impression outcomes in distal extension situations. Among the impression procedures evaluated, the selective pressure technique demonstrated slightly higher mean tissue displacement values, closely followed by the Hindels method, although the difference between these two techniques was not statistically significant. In contrast, the functional reline method exhibited comparatively lower tissue displacement. Collectively, these results suggest that impression techniques employing controlled pressure may contribute to more favorable tissue recording in distal extension removable partial denture cases; further investigations incorporating larger sample sizes and clinical outcome measures are warranted to substantiate these findings.
